# Foreign Body Implanted Into the Penis During Traffic Accident

**DOI:** 10.7759/cureus.39857

**Published:** 2023-06-02

**Authors:** Nihat Turkmen, Cemil Kutsal, Abdullah H Yavuzsan, Sinan L Kirecci

**Affiliations:** 1 Urology, University of Health Sciences, Sisli Hamidiye Etfal Training and Research Hospital, Istanbul, TUR

**Keywords:** genital trauma, penis, foreign body, erection, traffic accident

## Abstract

Although there are some reports describing foreign body implantation into the penis by intentional manipulation, no records about patients got aware of it many years after traffic accidents. A 29-year-old male patient had been severely injured in a traffic accident 13 years ago. Following a coma state for several months, he had no any symptom for a long time. Four years later, he got aware of the inconvenience on the ventral side of his penis during erection. His partner had also complained of pain during coitus. When he was admitted to our clinic, there was a semi-mobile, fibrous dense 2x2 cm knob on the ventral side of the penis consisting of a coronal sulcus. Under local anesthesia, we got out of a piece of glass. He was discharged after enough follow-up periods without complication. The interesting point of this case was not the clinical condition of the patient; it was that no one could consider a coma patient would have a complaint of penis injury several years later. This case showed us, one more time, how important the complete physical examination was.

## Introduction

Foreign bodies, such as glass or mica, being inserted into the body of the penis into the urethra, or under the skin of the penis have been reported in the literature, as well as broken needle tips remaining inside the penis following therapy [1-6]. In most such cases, the cause was intentional manipulation. However, in traffic accidents with multiorgan trauma, foreign bodies may find their way into different parts of the body, and in this paper, we present a case of a man who lived for 13 years with a piece of glass in the distal ventrum of his penis, which he acquired during a traffic accident.

## Case presentation

The 29-year-old male patient had a traffic accident 13 years ago. He was comatose for two months after the accident and received treatment for multiorgan trauma during that time. The patient reports memory loss for the next two years. He palpated a lump on the underside of his penis four years after the accident. He was complaining of pain during erection, and his partner was also uncomfortable during coitus.

**Figure 1:**
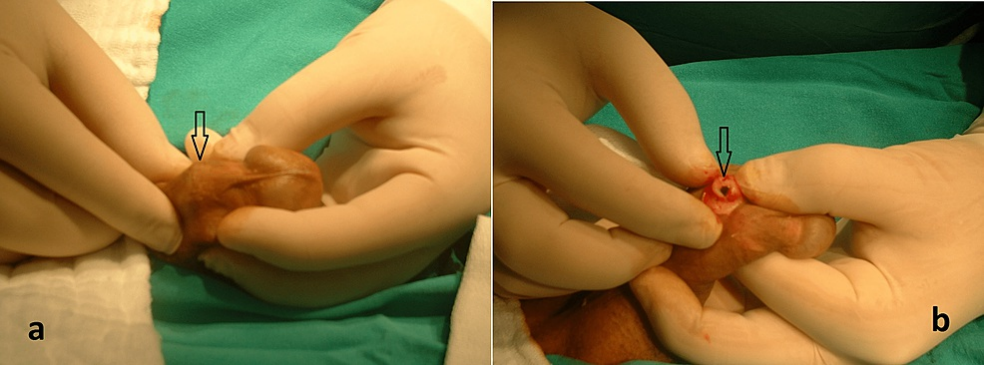
Intraoperative images before extracting the foreign body a. Palpation of the foreign body (arrow) before the incision; b. Image of reaching foreign body (arrow) after incision of the skin, subcutaneous, Colles' and Buck's fascia.

**Figure 2:**
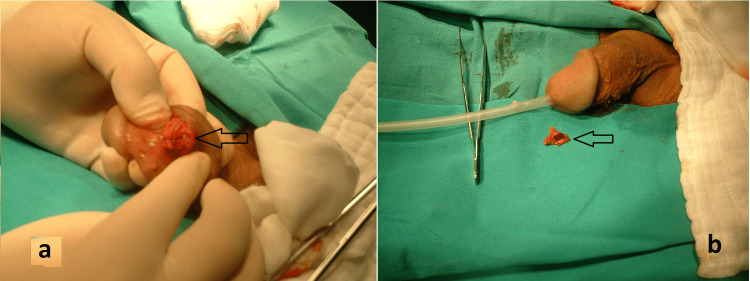
Intraoperative images after removal of the foreign body a. Image of the incisional area after removal of the foreign body (arrow), b. Image of the removed 2 cm piece of glass (arrow).

When he was admitted to our clinic, there was a hard, semi-mobile, palpable lump of approximately 2×2 cm located on the ventral side of the penis, encompassing the coronal sulcus (Figure 1a). The patient had no medical or surgical history of any disease or operation. He also did not have any urination problems. Then penile ultrasonography was applied to the patient. On ultrasonography, it was reported that there was a hyperechoic lesion with minimal vascularity around it. In the foreground, it was reported that this lesion might be a granuloma. Surgical exploration was decided because the patient had a lesion with vascularity on ultrasonography and a painful erection. Under local anesthesia, the skin, the underskin, and Colles’ and Buck’s fascia were passed with a circumferential incision. A 2×2 cm piece of glass, located adjacent to the tunica albuginea of the right cavernous body and the urethra (Figure 1b and Figures 2a, 2b), was observed and then extracted. He was discharged after a day of follow-up without any complications.

## Discussion

Many papers have reported foreign bodies in the penis and urethra. Needle tips broken during intracavernous injections [1,6], granulomas in occupational disease [2], intentionally inserting a plastic ball [4] or mica [5] under the skin, or insertion of a foreign body for masturbatory purposes [3] have all been described in different papers. However, all such papers mention intentional manipulation, and there have been no cases reporting a foreign body being implanted during a traffic accident.

## Conclusions

General body traumas, like in traffic accidents, may result in loss of consciousness, and thus other pathological conditions may be ignored while attending to life-threatening problems. In this case, the patient’s vegetative state concealed his penis injury. Faced with multiple body traumas and comas, the clinicians did not consider the penis to be a part of the body worth attending to. This case shows that every part of the body can be involved in multiple trauma and that a complete physical examination is very important.
